# Cellular Resilience as a Potential Predictor of Lifespan

**DOI:** 10.1093/geroni/igab046.620

**Published:** 2021-12-17

**Authors:** Adam Salmon

**Affiliations:** University of Texas Health San Antonio, San Antonio, Texas, United States

## Abstract

The progressive decline of resilience during the aging process across multiple functional systems suggests basic biological mechanisms of regulation. We exploited a primary cell model to identify markers of cellular resilience or the ability of cells in culture to respond and return to homeostasis following acute challenge including metabolic, oxidative, or proteostatic stress. Using primary fibroblasts from minimally-invasive skin biopsies of genetically heterogeneous mice, we are able to determine individual cellular resilience as well as the normal lifespan and healthspan of each donor. Our studies suggest donor age and sex affect cellular resilience and that this measure of resilience can predict functional outcomes in some interventional studies. While longevity studies continue, these studies point to a potential highly important marker of healthspan and longevity as well as a model to delineate the biology of resilience in animal and translational models.

